# Excessive TV viewing and cardiovascular disease risk factors in adolescents. The AVENA cross-sectional study

**DOI:** 10.1186/1471-2458-10-274

**Published:** 2010-05-25

**Authors:** David Martinez-Gomez, J Pablo Rey-López, Palma Chillón, Sonia Gómez-Martínez, Germán Vicente-Rodríguez, Miguel Martín-Matillas, Miguel Garcia-Fuentes, Manuel Delgado, Luis A Moreno, Oscar L Veiga, Joey C Eisenmann, Ascension Marcos, AVENA Study Group

**Affiliations:** 1 Immunonutrition Research Group, Department of Metabolism and Nutrition, Instituto del Frio, Institute of Food Science, Technology and Nutrition (ICTAN), Spanish National Research Council (CSIC), Madrid, Spain; 2 Department of Physical Education, Sport and Human Movement, Universidad Autónoma de Madrid, Madrid, Spain; 3 School of Health Sciences, University of Zaragoza, Zaragoza, Spain; 4 Department of Physical Education and Sport, School of Physical Activity and Sport Sciences, University of Granada, Granada, Spain; 5 Department of Pediatrics, University of Cantabria, Santander, Spain; 6 Department of Kinesiology and Pediatrics & Human Development, East Lansing, MI, USA

## Abstract

**Background:**

Excessive television (TV) viewing might play an important role in the development of cardiovascular disease (CVD). The aim of this study was to examine the independent associations between TV viewing and CVD risk factors in adolescents.

**Methods:**

A sample of 425 adolescents, aged 13- to 18.5-year-old, was included in this study. Body mass index (BMI), waist circumference (WC), glucose, total cholesterol, triglycerides, HDL-cholesterol, LDL-cholesterol, apolipoprotein (apo) A-1, apo B-100, and lipoprotein(a) levels were determined. A composite CVD risk score was computed based on age-, sex-, sexual maturation- and race-standardized triglycerides, HDL-cholesterol, LDL-cholesterol and glucose. TV viewing was self-reported.

**Results:**

Two hundred and twenty-five adolescents (53%) who spent >3 hrs/day watching TV were considered as the "high TV viewing" group. Ninety-nine adolescents (23%) from the total sample were classified as overweight according to International age- and sex-specific BMI values. The high TV viewing group had significantly less favorable values of HDL-cholesterol, glucose, apo A1 and CVD score, independent of age, sex, sexual maturation, race and weight status. There was a significant interaction effect of TV viewing × weight status (P = 0.002) on WC, and the negative influence of TV viewing on WC persisted in the overweight group (P = 0.031) but was attenuated in non-overweight adolescents (P > 0.05).

**Conclusion:**

Excessive TV viewing seems to be related to an unfavorable CVD risk factors profile in adolescence. Reducing TV viewing in overweight adolescents might be beneficial to decrease abdominal body fat.

## Background

Cardiovascular and metabolic diseases are the principal causes of mortality in developed countries [1]. Therefore, cardiovascular diseases (CVD) and metabolic risk factors are very important concerns in public health policies [2]. To prevent atherosclerosis, type 2 diabetes, and the metabolic syndrome, it is necessary to develop actions from childhood and adolescence because CVD risk factors track towards adulthood [3]. Likewise, the increasing prevalence of CVD risk factors in children and adolescents might be, at least in part, a response to the worldwide trends in pediatric overweight and obesity [4,5].

Adequate habitual physical activity and a healthy diet are the basic cornerstones to prevent obesity and CVD [6]. However, chronic diseases result from the interaction among many factors [7], and the evaluation of other lifestyles could be relevant. In the last decades, the development of technologies has been related to sedentary behavior and obesity in children and adolescents [8]. Nowadays, there is a social trend to mistakenly consider physical activity and sedentary behaviors as opposite poles from the same continuous (e.g. if a child does not achieve enough physical activity, he or she may be categorized as sedentary or couch potato), but studies in adolescents have shown that time spent in sedentary behaviors must be considered independently of physical activity [9], and hence, both sedentary behavior and physical activity may have a different effect in the prevention and development of CVD.

Television (TV) viewing is a well-known sedentary behavior and too much time spent watching TV is considered an unhealthy behavior associated with obesity and other harmful health outcomes in youth [10]. Little is known about how TV viewing and CVD risk factors are linked in youth because the majority of the studies have been conducted in adults [11-14]. To our knowledge, in children and adolescents, several studies have analyzed the associations between TV viewing and individual CVD risk factors-abdominal fat [15,16], lipid abnormalities [17,18], hypertension [19,20], insulin resistance [21]- and only two studies have examined the associations using composite CVD risk scores [22,23]. The majority of the above mentioned studies did not show whether these associations were independent of body fat.

On the other hand, interventional studies reducing TV viewing in apparently healthy children and adolescents have shown small or no significant effects on obesity [8,24], whereas interventions on obese children by decreasing time spent watching TV have shown a positive effect on obesity indicators [25,26]. Hence, these findings suggest that the effect of TV viewing on obesity might differ by weight status. Nevertheless, whether associations between TV viewing and CVD risks factors vary by weight status in adolescents have not been evaluated yet.

Therefore, the aim of the present study was to examine the independent association of excessive TV viewing with individual and clustered CVD risk factors in adolescents. Additionally, we also examined whether a different weight status modifies the associations between TV viewing and CVD risk factors.

## Methods

### Design and participants

The AVENA (Alimentación y Valoración del Estado Nutricional de los Adolescentes: Food and Assessment of the Nutritional Status of Spanish Adolescents) study is a cross-sectional and multicenter study performed in Spanish adolescents aged 13.0 to 18.5-years. Design and methodologies of the AVENA study have been previously described [27]. In brief, 2859 Spanish adolescents were assessed in 5 Spanish cities (Granada, Madrid, Murcia, Santander and Zaragoza) between 2000 and 2002. Health and lifestyle indicators, body composition, and health-related physical fitness were assessed in all adolescents. Moreover, in a subsample of 581 adolescents, blood samples were collected to determine hematological, lipid, immunological and genetic parameters. Of this subsample, a total of 214 boys and 211 girls (n = 425, 97% Caucasian) with complete and valid data on anthropometry measurements and self-reported time spent watching TV were included in the current study. Socioeconomic status (SES) was also parent-reported and defined by the educational achievement of the mother (elementary school, middle school, high school and university) but this information was available in 378 adolescents (89%). Parents and guardians were informed about the characteristics and aims of the study, and they gave their written informed consent. The AVENA study protocol was approved by the Review Committee for Research Involving Human Subjects from Marques de Valdecilla University Hospital (Santander, Spain).

### Physical examination

Height (m) and body weight (kg) were measured barefooted and wearing light underclothes. Height was measured to the nearest 1 mm and body weight to the nearest 0.05 kg by using a standard beam balance with a stadiometer. Body mass index (BMI) was calculated as body weight divided by height squared (kg/m^2^). Waist circumference (WC) was measured (cm) with a non-elastic tape to the nearest 1 mm between the lowest rib margin and the iliac crest, near the level of the umbilicus, at the end of gentle expiration. The anthropometric protocols in the AVENA study were previously harmonized and described elsewhere [28]. In the current study, overweight (including obesity) adolescents were classified according to age- and sex-specific cut offs proposed by the International Obesity Task Force [29]. At the time of the anthropometry measurements, sexual maturation of each adolescent was assessed (I to V) according to Tanner and Whitehouse [30]. The standard stages of sexual maturation describe breast and pubic hair development in adolescent girls, and genital and pubic hair development in adolescent boys.

### TV viewing

Adolescents' time (hrs/day) watching TV was assessed by questionnaire. Adolescents were asked as follows: How many hours do you usually spend watching TV per day? Adolescents had to select one of the following categories: 1) None 2) Less than 1/2 hour 3) Between 1/2-1 hour 4) Between 1-3 hours 5) Between 3-4 hours 6) More than 4 hours. Similarly to previous studies in adolescents in the AVENA framework [31], those adolescents who spent ≤3 hrs/day (categories 1 to 4) watching TV were considered as "low TV viewing", whereas adolescents who spent >3 hrs/day were classified as "high TV viewing" (categories 5 and 6).

### Blood sampling

After overnight fasting, blood samples were collected between 8:00 and 9:30 AM by venipuncture. Within 1-hr after blood collection, serum was separated by centrifugation and divided into aliquots. Triglycerides (TG), total cholesterol (TC), high-density lipoprotein cholesterol (HDL-C) and glucose were measured by enzymatic assay using a Hitachi 911 Analyzer (Roche Diagnostics, Indianapolis, Ind., USA). Low-density lipoprotein cholesterol (LDL-C) was calculated with the Friedewald formula: LDL-C = (TC-HDL-C) - (TG/5). Apolipoprotein (apo) A-I, apo B-100 and lipoprotein(a) levels were measured using a immunonephelometric assay on Array 306 system (Beckman GMI, Inc., Albertville, MN, USA). Quality control of the assays was assured by the Regional Health Authority, as compulsory for all clinical laboratories in Spain. A more detailed description of the blood analysis has been reported elsewhere [32].

### Continuous CVD risk score

A composite CVD risk score was created using TG, HDL-C, LDL-C, and glucose values. This CVD risk score including lipids and metabolic parameters has been used in a previous study with adolescents [33]. The four selected CVD factors were standardized by regressing them onto age, sex, sexual maturation and race variables. Once each CVD variable was regressed onto the independent variables, the standardized residual (Z-scores) was saved. Since the standardized HDL-C is inversely related to metabolic risk it was multiplied by -1. The standardized residuals were summed to create the CVD risk score denoting the higher score the less favorable CVD profile.

### Statistical analysis

The distribution of continuous variables was assessed for normality and natural-log transformations were performed when necessary. Data were described by mean ± SD unless otherwise stated. Differences between adolescent boys and girls were determined by one-way analysis of variance (ANOVA) for continuous variables and the Chi-square test for categorical data.

Differences between non-overweight and overweight groups for individual CVD risk factors (WC, TG, TC, HDL-C, LDL-C, Apo A-1, Apo B-100, and lipoprotein(a)) were assessed by analysis of covariance (ANCOVA) adjusted by age, sex, sexual maturation and race. Differences between weight status groups for the continuous CVD risk score were assessed by ANOVA because the variable was previously age-, sex-, sexual maturation-- and race-standardized.

Differences between TV viewing (low and high) groups for individuals CVD risk factors were assessed by ANCOVA adjusted by potential confounders whereas ANOVA was used for the continuous CVD risk score. To examine the independent associations between TV viewing and CVD risk factors, a second model was performed including weight status as a fixed factor. A final model was also performed including the interaction term TV viewing × weight status. The level of significance was set at p < 0.05 and analyses were carried out using SPSS (SPSS Inc., Chicago, IL, US) version 13.0 for Macintosh.

## Results

Table 1 provides an overview of the characteristics of the study participants by sex. Adolescent boys were taller and heavier than girls but there was no significant difference in BMI levels. Sixty adolescent boys (28%) and thirty-nine adolescent girls (19%) were classified as overweight. For the CVD risk factors, adolescent boys had significantly higher abdominal body fat, and less favorable values of TG, HDL-C, glucose, and Apo A-1 than girls. In contrast, adolescent girls had less favorable values of TC, LDL-C and Apo B-100 than boys. Two hundred and twenty-five adolescents (53%) were classified in the "high TV viewing" group and a significantly higher percentage of adolescent boys than girls were classified in the high TV viewing group (p = 0.023).

**Table 1:** Baseline characteristics of the sample (n = 425)

	Adolescent boys (n = 214)	Adolescent girls (n = 211)	p
Age (yrs)	14.9 ± 1.2	14.8 ± 1.4	0.370
Weight (kg)	64.4 ± 13.3	56.3 ± 10.6	**<0.001**
Height (m)	1.7 ± 0.1	1.6 ± 0.1	**<0.001**
Body mass index (kg/m^2^)	22.1 ± 3.9	21.6 ± 3.5	0.177
Non-overweight/Overweight	154/60	162/39	**0.020**
Waist circumference (cm)	77.1 ± 9.4	71.1 ± 8.4	**<0.001**
Sexual maturation (I/II/III/IV/V)	1/10/28/86/89	0/5/18/110/78	**<0.001**
Triglycerides (mg/dl)^§^	71.1 ± 31.8	65.1 ± 27.0	**0.037**
Total cholesterol (mg/dl)^§^	155.8 ± 26.3	170.1 ± 25.4	**<0.001**
HDL-cholesterol (mg/dl)^§^	51.4 ± 10.0	59.4 ± 11.8	**<0.001**
LDL-cholesterol (mg/dl)^§^	90.2 ± 23.5	97.8 ± 22.7	**0.001**
Glucose (mg/dl)^§^	95.4 ± 9.7	91.5 ± 8.4	**<0.001**
Apoliprotein-A1 (mg/dl)^§^	115.5 ± 15.5	125.5 ± 17.5	**<0.001**
Apoliprotein-B100 (mg/dl)^§^	65.9 ± 14.8	69.3 ± 13.4	**0.013**
Lipoprotein(a) (mg/dl)^§^	30.8 ± 36.8	31.6 ± 38.9	0.683
Low TV viewing/High TV viewing^†^	89/125	111/100	**0.023**

Values are mean ± SD. ^§^Values were natural log-transformed, but not transformed values are presented in the table. ^† ^High TV viewing: >3 hrs/day. Statistical analyses were analysis of variance for continuous variables and the Chi-square test for categorical variables.

Differences between weight status groups for CVD risk factors are shown in Table 2. Adolescents classified as overweight had less favorable values of WC, TG, HDL-C, LDL-C, Apo A-1, Apo B-100 than non-overweight adolescents controlling for age, sex, sexual maturation and race. Moreover, overweight adolescents also had a higher CVD risk score than non-overweight adolescents (p < 0.001).

**Table 2:** Differences in cardiovascular disease (CVD) risk factors between weight status groups among adolescents (n = 425)

	Non-overweight (n = 326)	Overweight^† ^(n = 99)	p
Waist circumference (cm)^‡^	72.0 ± 2.1	86.5 ± 2.2	**<0.001**
Triglycerides (mg/dl)^‡§^	63.0 ± 9.4	71.7 ± 9.9	**0.011**
Total cholesterol (mg/dl)^‡§^	167.1 ± 8.2	169.3 ± 8.7	0.462
HDL-cholesterol (mg/dl)^‡§^	61.5 ± 3.3	55.7 ± 3.6	**<0.001**
LDL-cholesterol (mg/dl)^‡§^	93.0 ± 7.2	99.3 ± 7.8	**0.019**
Glucose (mg/dl)^‡§^	91.9 ± 2.8	93.6 ± 3.0	0.109
Apoliprotein-A1 (mg/dl)^‡§^	124.0 ± 5.0	119.1 ± 5.5	**0.003**
Apoliprotein-B100 (mg/dl)^‡§^	65.7 ± 4.5	69.5 ± 4.7	**0.021**
Lipoprotein(a) (mg/dl)^‡§^	30.1 ± 2.0	34.8 ± 4.3	0.437
CVD risk score^#^	-0.9 ± 0.7	0.4 ± 0.7	**<0.001**

Values are mean ± SE. ^† ^Including obesity. ^‡ ^Analysis of covariance adjusted by age, sex, sexual maturation and race. ^§ ^Values were natural log-transformed, but not transformed values are presented in the table. ^# ^Analysis of variance.

Differences between TV viewing groups for CVD risk factors are shown in Table 3. Adolescents in the high TV viewing group had less favorable values of HDL-C, glucose and Apo A-1, controlling for potential confounders, as well as a lower CVD risk score (p < 0.001). When weight status was also included into the model as a confounder variable (model 2), the main results did not change (Table 3).

**Table 3:** Differences in cardiovascular disease (CVD) risk factors between TV viewing groups^† ^among adolescents (n = 425)

		Low TV viewing (n = 200)	High TV viewing (n = 225)	p
Waist circumference (cm)	Model 1	72.2 ± 2.9	73.2 ± 2.9	0.259
	Model 2	78.8 ± 2.1	79.5 ± 2.1	0.299
				
Triglycerides (mg/dl)^§^	Model 1	60.3 ± 9.6	65.3 ± 9.4	0.085
	Model 2	64.1 ± 9.7	69.0 ± 9.5	0.096
				
Total cholesterol (mg/dl)^§^	Model 1	166.8 ± 8.4	167.5 ± 8.3	0.809
	Model 2	167.8 ± 8.6	168.4 ± 8.4	0.824
				
HDL-cholesterol (mg/dl)^§^	Model 1	63.2 ± 3.5	60.1 ± 3.4	**0.004**
	Model 2	60.6 ± 3.5	57.6 ± 3.4	**0.005**
				
LDL-cholesterol (mg/dl)^§^	Model 1	91.6 ± 7.5	94.3 ± 7.4	0.242
	Model 2	94.4 ± 7.6	97.0 ± 7.5	0.265
				
Glucose (mg/dl)^§^	Model 1	89.7 ± 2.8	93.2 ± 2.8	**<0.001**
	Model 2	90.4 ± 2.9	93.9 ± 2.8	**<0.001**
				
Apoliprotein-A1 (mg/dl)^§^	Model 1	127.7 ± 5.3	122.7 ± 5.2	**0.002**
	Model 2	125.2 ± 5.3	120.4 ± 5.2	**0.002**
				
Apoliprotein-B100 (mg/dl)^§^	Model 1	65.0 ± 4.6	66.4 ± 4.5	0.337
	Model 2	66.7 ± 4.6	68.0 ± 4.6	0.366
				
Lipoprotein(a) (mg/dl)^§^	Model 1	26.5 ± 12.3	27.1 ± 12.2	0.876
	Model 2	28.9 ± 12.5	29.3 ± 12.4	0.900
				
CVD risk score^‡^	Model 1	-1.5 ± 0.7	-0.5 ± 0.7	**<0.001**
	Model 2	-0.9 ± 0.7	0.0 ± 0.7	**<0.001**

Values are mean ± SE. ^† ^High TV viewing: >3 hrs/day.

Model 1: Analysis of covariance adjusted by age, sex, sexual maturation and race. Model 2: Analysis of covariance adjusted by model 1 + weight status. ^§ ^Values were natural log-transformed, but not transformed values are presented in the table. ^‡ ^Analysis of variance in Model 1 and analysis of variance adjusted by weight status in Model 2.

**Figure 1:**
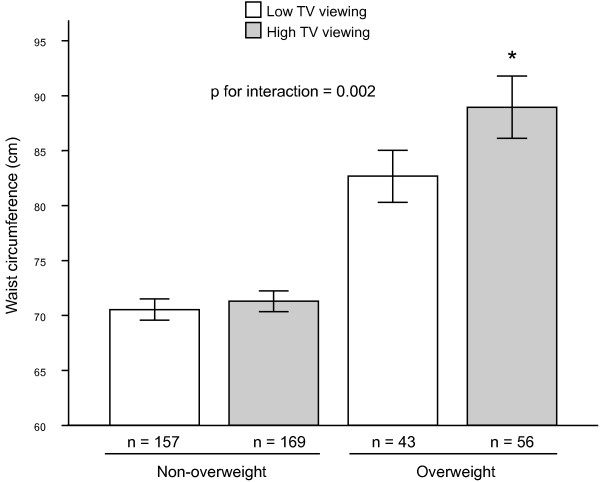
**Differences in waist circumference across weight status and TV viewing groups among adolescents (n = 425)**. Values are mean ± SE. High TV viewing: >3 hrs/day. Analyses of covariance adjusted by age, sex, sexual maturation and race. * p = 0.031 compared with the overweight/low TV viewing group.

Finally, we analyzed whether the influence of TV viewing on CVD risk factors differ by weight status including a TV viewing × weight status interaction term into the model (model 3). These analyses showed that differences between TV viewing groups for CVD risk factors remained significant, but there was a significant interaction effect of TV viewing × weight status on WC (p = 0.002). Differences in WC across TV viewing and weight status groups in the adolescent sample are illustrated in Figure 1. Thus, the influence of TV viewing on WC in adolescents was only significant in the overweight group (p = 0.031), whereas this influence was attenuated in the non-overweight group (p > 0.05). All the results did not change when analyses were adjusted for SES as a confounder variable (data not shown).

## Discussion

The main results of our study suggest that excessive TV viewing is positively associated with CVD risk factors such as HDL-C, glucose, Apo A-1 and a continuous CVD risk score in adolescents, independently of potential confounders including weight status. These findings provide some evidence regarding the detrimental role of excessive TV viewing on CVD risk factors development in adolescence. In addition, further analyses revealed that weight status might modify the influence of TV viewing on abdominal body fat. Thus, the negative influence of TV viewing on WC remained in the overweight group but was attenuated in the non-overweight group.

Several studies in adults have shown similar findings to those found in the current study. In these studies the time spent watching TV has been positively associated with CVD risk factors [11-14]. For example, Healy et al. [17] showed a detrimental dose-response association of TV viewing with WC, systolic blood pressure and 2-h plasma glucose in 4064 Australian adults [17]. Recently, Dustan et al. [34] have shown that excessive TV viewing is associated with increased risk of all-cause and CVD mortality in 8800 Australian adults. Similar results were found in a men sample where the combination of time riding in a car and time spent watching TV were positively associated with CVD death [35].

Despite of these findings in adult populations, the evidence linking TV viewing to CVD risk factors in children and adolescents is limited. For example, positive associations between TV viewing and abdominal body fat have been found in cross-sectional studies [15,16]. Nevertheless, several reviews have stated the weakness of these findings because poor results have been found in longitudinal and interventional studies [8]. Significant associations between TV viewing and blood markers (TC, insulin and glucose) [17,18] and blood pressure have also been found in children and adolescents [19,20]. However, only in some cases body fat indicators were included as a confounder variable into analyses. For this reason, we cannot know whether the influence of TV viewing on CVD risk factors is independent of body fat-direct role- or whether body fat plays a pivotal role between TV viewing and a less favorable CVD risk factor profile-indirect role.

In the European Youth Heart Study, Ekelund *et al*. [22] reported an independent association between TV viewing and metabolic syndrome risk factors in 1921 children and adolescents from 3 regions in Europe (Denmark, Estonia, and Portugal), but this association was mediated by adiposity (sum of 4 skinfold-thicknesses). Hence, the authors concluded the importance of reducing the time spent watching TV among children and adolescents in order to reduce directly body fat, and indirectly CVD risk factors. On the contrary, the associations between TV viewing and several CVD risk factors in the current study were not mediated by weight status. Interestingly, we also found that the influence of TV viewing on WC varied by weight status. The detrimental influence of TV viewing was maintained in the overweight group but not in the non-overweight group. These results suggest that reducing TV viewing time in overweight adolescents may have a beneficial influence on abdominal body fat. This interesting finding might explain the mixed effects found in intervention studies that decrease sedentary behaviors in apparently healthy and overweight children and adolescent [8,24-26].

TV viewing is commonly used as a proxy to describe sedentary behavior even though the capacity to describe sedentary time using this approach is constantly questioned [36,37]. New research directions have used objective methods to assess daytime sedentary patterns and several reports have suggested the use of accelerometry for these purposes [38]. Assessments using objective measures follow the idea of "inactivity physiology" posited by Hamilton *et al*. [39]. In order to observe the differences between both methods, we have previously assessed *sedentariness *using sedentary time by accelerometry, and TV viewing and computer use by parent-report in a 3- to 8-year-old sample [20]. On average, children spent 5 hrs/day in sedentary time and 1.5 hrs/day in screen time (TV + computer). These results show large differences between both methods. We consider that sedentary behavior with or without technology-mainly sitting time- and sedentary behavior using new technologies -mainly TV viewing- must be considered independently each other because both may be important in the development and prevention of CVD.

TV viewing in children and adolescents is usually associated with unhealthy behaviors. Firstly, TV viewing is associated with high consumption of soft drinks, salt, snacks, fat and low fruit and vegetable consumption [40]. Secondly, TV viewing may contribute to the development of sleep problems from adolescence to adulthood [41]. Thirdly, TV viewing may restrict the possibility of youth to participate in physical activity [42]. Fourthly, TV viewing contributes to inactive physiology as mentioned above. Hence, when reduction of time watching TV is suggested to prevent obesity and CVD risk factors, we may have influence on diet, sleep, physical activity and physical inactivity patterns that occur concurrently with TV viewing. Therefore, it is necessary to develop actions to reduce time watching TV from an early age. For children and adolescents, the American Academy of Pediatric recommends limits to no more than 2 hours per day of sedentary technologies, especially TV viewing [10]. Similarly, the Healthy People 2010 initiative promoted by the U.S. Department of Health and Human Services included the following objective: "*to increase the proportion of adolescents who view television 2 or fewer hours on a school day*" http://www.healthypeople.gov. As other authors [43,44], we could not use the 2 hours per day cut-off point. In our study, more than 50% adolescents spent >3 hrs/day watching TV, which is a similar prevalence to that found in a previous study in adolescents [44].

Results found in our study confirm that an overweight status is positively associated with individual (WC, TG, HDL-C, LDL-C, apo A-1, apo B-100) and clustered CVD risk factors in adolescents. Therefore, a special focus of attention must be aimed at overweight adolescents who view too much TV. Moreover, future studies are encouraged to evaluate more health indicators in addition to obesity outcomes (e.g. BMI, % body fat, WC) because the evidence is scarce with other CVD risk factors. For example, in addition to the traditional CVD risk factors related to the metabolic syndrome, we have analyzed the associations of TV viewing with Apo A-1, Apo B-100, and lipoprotein(a). Thus, our results suggest that Apo A-1 is inversely and independently associated with TV viewing. Recently, childhood and adolescence Apo A-1 levels have been considered predictors of subclinical atherosclerosis in adulthood [45].

Several limitations must be also mentioned in the current study. Our results are limited due to its cross-sectional design and causal directionality cannot be inferred. Moreover, one blood sample was used and that might not accurately reproduce long-term lipid and metabolic abnormalities. Unfortunately, blood pressure was not available in the AVENA Study, and therefore, we cannot compare findings from previous studies. TV viewing was measured using a single-response item. This type of questions to assess TV viewing has been widely used in large sample studies because objective measurements (direct observation, video, TV time manager) are not usually feasible in population studies. In children and adolescents, an adequate reliability and acceptable validity of these single 1-item questions to assess TV viewing have been observed [46]. In spite of this, single-response questions are preferred rather than combined questions (e.g. How many hours do you usually spend watching TV and using computer per day?) because computer use and video game play may have a different effect on metabolic and physiologic parameters [47]. Finally, we did not assess dietary patterns during TV viewing. Ekelund *et al*. [22] found that associations between TV viewing and CVD risk factors and adiposity were attenuated when dietary behavior while viewing TV was controlled into the model although results were not shown.

## Conclusions

Excessive TV viewing might aggravate several CVD risk factors in adolescents independently of their weight status. Furthermore, reducing the time spent watching TV might improve abdominal body fat in overweight adolescents. Specific intervention strategies are necessary in overweight adolescents who spent too much time daily watching TV. Experimental studies examining the role of TV viewing on traditional and new CVD risk factors in children and adolescents are warranted.

## Competing interests

The authors declare that they have no competing interests.

## Authors' contributions

Statistical analysis: DMG; Draft the manuscript: DMG; Funding and overall concept and design: AM, LAM, MGF and MD. Interpretation and acquisition of data: DMG, JPRL, PC, SGM, GVR, MMM, MGF, MD, LAM, OLV, JCE and AM. All authors read and approved the final manuscript.

## Pre-publication history

The pre-publication history for this paper can be accessed here:

http://www.biomedcentral.com/1471-2458/10/274/prepub
